# 9.N. Workshop: It Takes Two To Improve European Child Health (Care)

**DOI:** 10.1093/eurpub/ckac129.603

**Published:** 2022-10-25

## Abstract

*This abstract has been withdrawn*.

